# Emergency preparedness, resilience and response guidance for UK hospital transfusion teams

**DOI:** 10.1111/tme.12665

**Published:** 2020-02-04

**Authors:** Heidi Doughty, Fateha Chowdhury, the National Blood Transfusion Committee Emergency Planning Working Group, V Ameh, N Batrick, L Baxter, P Bolton–Maggs, T Cowdrey, S Glasgow, A Jackson, S Robinson, M Smith, J Staves, Y Sorour, K Pendry, J Uprichard, A Weaver, C Wilkes, S Allard, M Murphy, C. Kendrick, H Doughty, J Earley, L Frith

**Affiliations:** ^1^ NHS Blood and Transplant Clinical Services, Birmingham United Kingdom; ^2^ Imperial College Healthcare NHS Trust London United Kingdom

**Keywords:** emergency planning, major incidents, transfusion

## Abstract

**Objectives:**

To present Emergency Preparedness, Resilience and Response (EPRR) guidance for Hospital Transfusion Teams on behalf of the National Blood Transfusion Committee emergency planning working group.

**Background:**

The Civil Contingencies Act 2004 requires healthcare organisations to demonstrate that they can deal with major incidents while maintaining critical services. Recent mass casualty events and the use of transfusion‐based resuscitation have highlighted the evolving role of the Hospital Transfusion Team.

**Methods:**

This multi‐disciplinary advice is informed by recent global and national experience, the 2018 NHS England clinical guidelines for Major Incidents, and stakeholder workshops.

**Guidance:**

Transfusion staff should be familiar with local EPRR plans including casualty type and numbers. Staff should be exercised as part of wider Trust preparation, with documented roles and responsibilities. Transfusion support should be proactive and include blood issue, regulatory compliance and sample handling. Robust LIMS‐compatible emergency identification systems are essential to minimise errors. Emergency stock management requires rapid assessment of existing stock and estimated demand before re‐ordering. Initial demand should be based on 2 to 4 red blood cells (RBC) per patient admitted. Patients with significant haemorrhage may require further red cells and early haemostatic support. Where “universal” components are demanded, they should be gender appropriate. Senior staff should lead the response, log and communicate key decisions, and prepare for post‐incident recovery.

**Conclusions:**

Transfusion teams have an important role in ensuring continuity of transfusion support. Teams should develop their EPRR plans based on local plans and national guidance. Emergency preparedness should include post‐incident debriefing for ongoing staff support and future service improvement.

## 1 BACKGROUND

The Civil Contingencies Act 2004^1^ requires NHS organisations and the providers of NHS funded care to demonstrate that they can deal with major incidents while maintaining critical services. The healthcare community refers to this national programme of work as emergency preparedness, resilience and response (EPRR).^2^ The programme is overseen locally by the NHS England regional EPRR teams and informs transfusion preparedness. The National Blood Transfusion Committee (NBTC) Emergency Planning Working Group was originally set up in 2005 as a short‐lived working group with representation from Hospital Transfusion Laboratories, Emergency Departments (EDs) and NHS Blood and Transplant (NHSBT). The objective was to review the lessons identified following the July 7th London Bombings in 2005 and provide guidance. The main aims were meeting a potential surge in demand for blood components and to optimise Hospital Transfusion Laboratory support following a Mass Casualty Event (MCE). In 2017, there were several major incidents in the UK, which together with global events, have highlighted the need to revisit national transfusion emergency preparedness.^3^ The group was reconvened in late 2017.

In 2018, new clinical guidelines for use in Major Incidents and MCE were developed by NHS England following a period of multiple incidents.^4^ The incidents had presented a range of challenging clinical scenarios, such as blast injury and penetrating injury, unlike those seen in day‐to‐day practice. The organisational guidance and care pathways were developed to establish and share best practice using recent experience from both military and civilian healthcare. Transfusion support is now recognised as an essential element of modern trauma resuscitation with a move to earlier use of blood in major trauma.^5^ The primary purpose of this document is to provide revised guidance for Hospital Transfusion Teams to prepare for, and respond to, conventional Major Incidents and MCEs. It is designed to complement the NHS England clinical guidelines. However, other emergencies such as wide‐scale disruption of computer services and adverse weather may also significantly disrupt transfusion laboratory function and necessitate a broadening of scope. The guidance therefore considers the transfusion EPRR for both Major Incidents and large‐scale disruption due to other causes.

## 2 METHODOLOGY

### 2.1 *Working group and writing process*

The multi‐disciplinary working group was selected to represent the wider transfusion community together with academics and key users with recent experience of major incidents. The final guidance format was based on the framework of an existing hospital‐based plan and further developed using feedback from Regional Transfusion Committees' study days and workshops. The guidance is designed to provide the foundation for local policy and practice.

### 2.2 *Review of the manuscript*

The review of the manuscript was performed by members of the NBTC. The NBTC is accountable to the National Medical Director of NHS England through the Chief Scientific Officer. Membership includes Royal Colleges, specialist societies and other professional organisations, Chairs of the Regional Transfusion Committees and patient representatives. It has also been reviewed by various groups within NHSBT and representatives of the other UK Blood Transfusion Services. These organisations have commented but do not necessarily approve or endorse the contents.

## 3 DEFINITIONS

Each healthcare organisation should have in place plans for Major Incidents, Mass Casualty Incidents (MCIs), Critical Incidents and Business Continuity Incidents. Definitions for each may vary but the following definitions may be useful:

- Major Incident (MI): Any occurrence that presents a serious threat to the health of the community, disruption to service, or causes (or is likely to cause) such numbers or types of casualties as to require special arrangements to be implemented.
- MCI/MCE: NHS England defines an MCI for the health services as an incident (or series of incidents) causing casualties, on a scale that is beyond the normal resources of the emergency and healthcare services ability to manage.
- Business Continuity Incident: An event or occurrence that disrupts or might disrupt normal service delivery to below acceptable predefined levels, requiring special arrangements to be implemented until services can return to an acceptable level.

The decision to activate any pre‐prepared incident plan should follow a decision‐making framework. The triggers for activation and escalation will depend on the nature of the incident and the capability and capacity of the healthcare organisation.

## 4 EMERGENCY PREPAREDNESS

Hospital Trusts must include the Pathology Department and the Hospital Transfusion Team in Major Incident planning. The role of the Transfusion Team is to provide transfusion support throughout the incident within their Trust to both optimise patient care and make best use of resources.

NHSBT is responsible for ensuring the supply of critical biological products, including blood, together with related clinical services. NHSBT acts as a Category 1 responder with authority to enable blue‐light vehicles to make emergency deliveries. See resilience and mutual aid section.

The Ambulance Service is the lead for the delivery of pre‐hospital healthcare. It is unlikely that blood will be used at scene during large‐scale incidents, where the priority is rapid triage and transport of casualties. However, Ambulance Services should have arrangements with pre‐selected Transfusion Laboratories for the provision of blood to scene in Major Incidents.

Recent events have demonstrated the value of proactively using haematology/transfusion/pathology staff to move “forward” in the care pathway during a Major Incident, especially where there are large numbers of casualties. Staff are best placed where most transfusion samples are being collected and transfusion is taking place. Examples include the ED, resuscitation areas and operating theatres.

Members of the extended transfusion team may be used to assist in a range of supporting activities,^6^ including: transfusion triage, emergency issue of blood, the handling of blood samples and communication with the laboratory. The use of staff will be dependent on staffing levels and trust configuration together with individuals' background and experience. All staff should be appropriately trained, competed and rehearsed for their role.

Hospital transfusion laboratories may consider moving stocks of blood components to key clinical areas for use in a Major Incident. Where blood is moved, secure systems should be in place for blood selection, maintaining the cold chain and traceability records.

Trust identification badges must be worn by those staff attending any hospital in the event of a Major Incident or activation of the MCE plan. Consideration should be given to the additional identification of transfusion/haematology staff, such as the use of tabards, when deployed to incident management areas.

Each ward and department should hold their own action cards, ensuring that they are readily available and give key instructions of what is expected of staff during a Major Incident. The consultant with responsibility for transfusion and the transfusion laboratory manager are responsible for maintaining their own departmental action cards. All members of staff are responsible for knowing the content of the action card that relates to their role in a Major Incident.

Hospital Transfusion Laboratories should be aware of their Trust's pre‐determined casualty plan in the context of MCE. The casualty regulation and capability charts can be found within the NHS England Regional Incident Response plan. The anticipated casualty numbers within the plan should be used to determine normal bloodstock and consumable holdings, stock redistribution and replenishment.

All hospital departments should be aware of their Major Incident plans and be trained and exercised together. The Transfusion Team should be notified of appropriate local training events and exercises by their Trusts. Additional training may be available from a range of sources including NHS England and NHSBT.

## 5 INCIDENT NOTIFICATION AND COMMUNICATION

Incidents may be notified from internal or external sources. The traditional method for response activation is the Ambulance Service notifying ED via a designated hotline or switchboard; ED will then activate the Major Incident or Mass Casualty plan. The activation of emergency plans may activate security measures controlling access and exit from the site, resulting in a “Lockdown.” Trust Identification badges must be worn by all staff attending hospitals in “Lockdown” to gain timely access.

The initial internal communication cascade or call‐out list should include the Transfusion Laboratory, at all stages of the communication process from standby to stand‐down. Consideration should be given to the further cascade of information.

Hospital Transfusion Laboratories are currently advised to inform the Hospital Services department of the local NHSBT centre/stock holding unit once the hospital has been notified of a Major Incident and again when stood down. Hospital Services will then initiate NHSBT's internal Emergency Planning procedures.

Telephone communications may fail or be unreliable during a Major Incident. Trusts should have protocols for alternative means of internal and external communication in the event of a failure of traditional or digital telecommunication technology. Methods include the use of runners, walkie‐talkies, emergency phones, text messaging and other digital applications.

External communications are normally the responsibility of each Trust's strategic command. However, Hospital Transfusion Laboratories should be permitted to maintain ongoing communication with NHSBT. It is recommended that hospitals consider retaining external phone lines for communication as a resilience measure.

Press enquiries should be referred to the Trust's Press Liaison Officer. All communications for potential blood donors should be led by NHSBT. Blood donors will be encouraged to keep their future donation appointments to replenish stock and maintain the continuity of supply.

## 6 EMERGENCY RESPONSE

### 6.1 *Hospital transfusion laboratory response on notification*

A senior member of the Hospital Transfusion Laboratory should assume responsibility for transfusion services and assess the required response. A log should be started to record key decisions and handovers of senior staff and shifts. The following key areas should be considered.

#### 6.1.1 Staffing

An initial assessment of current laboratory staffing should be undertaken along with determining the need for additional personnel. Other transfusion staff should be redeployed according to departmental plans. Off‐duty staff should not report for duty until advised to do so. Staff reporting for work should use the pre‐determined hospital check‐in points according to Trust plans.

#### 6.1.2 Blood stock and critical consumables

Stock levels of blood components within the laboratory and in remote fridges, that is, ED, theatres and satellite fridges should immediately be assessed, as should the availability of other critical consumables, including reagents and transport containers. Stock levels should be enough to maintain business continuity during and after the event. The total available internal bloodstocks should be quickly determined before arranging additional supplies from NHSBT.

**Figure 1:**
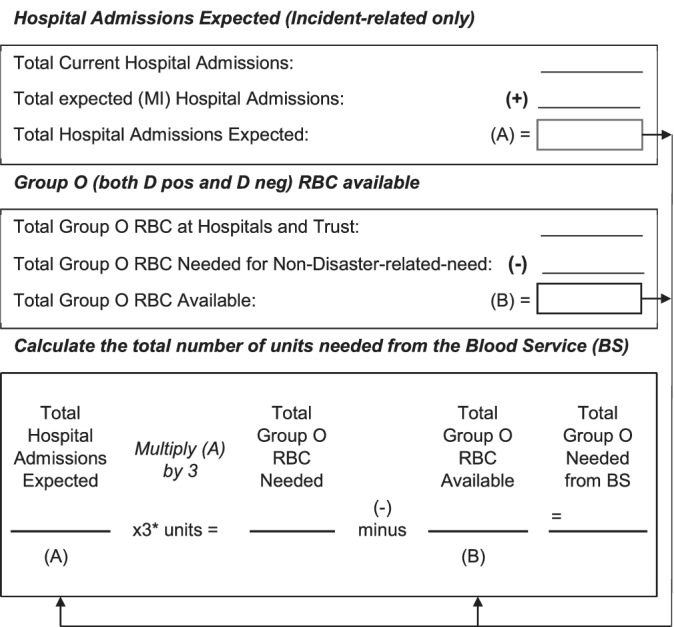
Blood ordering for major incidents: estimation of blood required. Calculator based on the assessment tool in the American Association of Blood Banks (2008) Disaster Operations Handbook. *Sources: American Association of Blood Banks (2008); Glasgow et al, 2013; Ramsey, 2017

The priority component required is expected to be Group O red cells. It is assumed that approximately 3 units are required per patient admitted with trauma.^7^ The initial blood order should take into consideration the hospital admissions expected, the total stock of group O already held, and the group O needed for non‐disaster related needs. An example of a calculator to estimate the requirement is shown in Figure 1.

#### 6.1.3 Stock movement

Trusts should initiate the movement and discharge of patients to receiving areas and create capacity for the reception of patients from the incident to ED, theatres and critical care areas. Routine surgery and some day care patient activity may be suspended. Blood already issued may no longer be immediately required for those cases. Consideration should be undertaken to de‐reserve and re‐centralise blood before re‐issuing to emergency areas to meet the potential surge in demand.

#### 6.1.4 Plasma

It is assumed that Trusts will hold enough frozen blood components to meet their planned admissions for the first hour. Plasma may be pre‐thawed and stored for 5 days for use in traumatic haemorrhage; however, cryoprecipitate is rarely routinely pre‐thawed. Hospitals that do not routinely use pre‐thawed plasma may wish to have procedures and training in place to enable staff to pre‐thaw plasma in preparation for urgent issue.

#### 6.1.5 Platelets

Early consideration should be given to the demand and storage for platelets, especially if Trusts are located some distance from NHSBT and do not routinely stock platelets. However, the current literature suggests that platelets are rarely routinely required in MCEs, except for the most severely injured patients.

#### 6.1.6 Pre‐hospital transfusion

In the context of Major Incidents, Transfusion Laboratories should anticipate the requirement for pre‐hospital transfusion and the implications for blood stock management.

#### 6.1.7 Blood service

NHSBT will respond to hospital orders from its national pre‐donated bloodstock. Current planning anticipates that several hospitals may order blood from the same stock holding unit/blood centre following an incident. It is assumed that most blood will be ordered as universal components and used within the first 6 hours. However, some patients may have an ongoing demand for blood, especially where repeat surgery may be necessary.

#### 6.1.8 Documentation

Major Incidents may be caused by criminal acts and are likely to be subject to subsequent investigations. All key decisions should be documented, and all documentation should be clear, accurate and timely. All documentation (electronic and paperwork) must be preserved. White boards should be photographed before cleaning. No material or details should be shared with unauthorised persons.

### 6.2 *The hospital response to a major incident*

Each hospital on activation should initiate their local command and control arrangements, including a central control room, to manage the overall response to the incident. The initial information available may be incomplete but situational understanding should evolve over time. Factors to be considered when determining the overall response include:

- Type of incident
- Type of injuries
- Anticipated number of patients
- Anticipated duration of the incident response
- Anticipated role of the support departments
- Status of the hospital and its ability to maintain business as usual

In most Trusts, all Major Incident patients will be received directly by ED. Patients should be triaged and treated as necessary by the ED staff supported by “surgical triage.” Patients requiring admission are commonly sent to Surgical Emergency Units where ongoing assessment and treatment are undertaken. Children requiring admission from the incident should be sent to a children's ward or designated area. Dependent on their injuries, some patients may be sent directly to theatres or Intensive Care Units (ICUs).

All patients seen in ED related to the incident should be documented. Staff in reception are responsible for recording the demographic details as incident patients arrive in ED. Most Trusts will set up a temporary reception desk. A pre‐numbered casualty card should be issued to each incident patient, together with bar coded identification bands and labels.

Guidelines for identifying “unknown” patients in emergency and mass casualty situations recommend *nonsequential* unique patient identifiers and gender as a minimum requirement. This is particularly important if several unknown patients are admitted together. All samples, whether from known or unknown patients, should also include the date and time of sampling and signature of the person taking that sample.^8^

When patients are admitted, the patient administration system, or manual equivalent, must be used to enter the patient as an admission, noting that they are part of a Major Incident.

## 7 TRANSFUSION SAFETY AND COMPONENT SELECTION

### 7.1 *Patient identification and blood samples*

The biggest transfusion risk in the context of Major Incidents is the accidental transfusion of ABO incompatible blood due to misidentification (see The hospital response to a major incident section). The 2018 Patient Safety Alert has provided further guidance for temporary identification to accommodate hospital transfers, which cover names, temporary numbers and options for indicating age.^9^ It is recommended that Transfusion teams discuss this alert and have local clinical agreements in place, which are compatible with their LIMS.

Baseline blood samples for pre‐transfusion testing should be obtained before administration of any blood components. A second confirmatory sample for transfusion should be taken as soon as possible and labelled independently from the first sample to confidently determine the patient's ABO and D group.^10^

The use of group‐specific blood is normally recommended once the patient's blood group has been confirmed. There are advantages both to the individual patient and the wider population if the patient can be safely transfused with group‐specific blood components. However, it is recognised that in some chaotic situations this may not be possible, and the initial use of group O blood may be the safest option.

The gender of the patient should be included on both the blood sample bottles and request forms to optimise blood group selection. Individuals of childbearing potential are at risk of developing atypical antibodies following blood transfusion which may harm future pregnancies. These patients therefore should receive D negative and K negative red cells. Pathology disciplines should default to the female gender in the event of an unidentified casualty where the gender has not been specified.

Request forms should include treatment priority, age or estimated age and special requirements if known. Distinguishing children from adults enables age‐related criteria to be applied to component selection. In addition, the treatment priority may influence the timely selection of component substitutions (see selection and issue of blood components section) or alternatives to transfusion (see guidance for clinical blood use section). Additional details associated with the request for transfusion testing should include any recent transfusion, including pre‐hospital transfusion and blood use in other treatment facilities.

There should be clear guidelines regarding the change from the Major Incident identifier to the routine hospital identifier, particularly in relation to transfusion samples. In most Trusts, this change takes place after the immediate resuscitation and surgical phase, that is, once the patient is clinically stable in ICU. A new “group and save” transfusion blood sample and a second confirmatory sample will be required using the new number.

### 7.2 *Guidance for clinical blood use*

Major Incidents may not result in many casualties with traumatic haemorrhage. In incidents classed as MCEs, current national EPRR planning is based on triage systems in which Priority 1 (P1) require immediate life‐saving intervention, P2 require intervention that could be delayed, and P3 are “walking wounded” or with minor injuries.^4^, ^11^ It is anticipated that only the P1s and P2s will require admission to hospital however priorities may change.

Reviews of past MCEs recommend planning for a red cell demand of 2 to 4 units for each casualty admitted with bleeding.^12^, ^13^, ^14^ An early estimate for more severely injured casualties admitted to trauma centres, that is, P1 casualties with massive haemorrhage, is near 6 units of RBC in 24 hours.^15^ Many of these patients may also require plasma, platelets and cryoprecipitate as guided by Major Haemorrhage protocols.^8^

Trusts should ensure that they have a policy for the management of massive haemorrhage and massive transfusion and this should be incorporated into the Major Incident plan in order to promote prompt and appropriate use of haemostatic blood components in this setting.^16^

Transfusion support should be optimised using the principles of *Patient Blood Management*. The two pillars most relevant to the immediate care of the patient with traumatic haemorrhage are: minimise blood loss, that is, haemorrhage control together with tranexamic acid and tolerance of anaemia. Measures to “optimise red cell mass” using iron (oral or intravenous) may be considered in the post‐operative phase.

Definitive haemorrhage control often requires surgery. Patients may require repeat surgery. Trusts should consider having an Intra‐Operative Cell Salvage service for use in major haemorrhage, including traumatic haemorrhage to reduce reliance on allogeneic blood.

Trusts should have contingency plans for major blood shortages incorporated into Major Incident plans. National integrated blood shortage plans include guidance for the clinical prioritisation of red cells^17^ and platelets.^18^

### 7.3 *Selection and issue of blood components*

All patients admitted to hospital should have a baseline sample taken for transfusion testing of blood group (ABO and D) and atypical antibody screen. However, blood grouping should be initially prioritised to the most urgent cases (P1 and P2 cases), that is, those who are bleeding and most likely to require blood components. Laboratory procedures should be in place to prioritise and handle emergency samples.

Samples from patients receiving ABO nonidentical blood may subsequently show two populations of cells during blood grouping. For example, if multiple Group O red cells are transfused into in a non‐O patient, this could make it difficult to obtain a clear ABO group. Group O red cells should be issued where there is uncertainty. In addition, such patients may not be eligible for subsequent electronic issue of blood and alternative arrangements must be in place for the timely release of blood components.

It is anticipated that “universal components” may be used where the ABO and D groups are unknown. Appropriate blood group substitutions should be considered to optimise stock management of all blood components. An example is the use of group A (High Titre neg) plasma as a substitute for group AB plasma.^8^

Group O positive red cells may need to be used in unknown males.^19^ D and K negative blood should be prioritised for unknown females under the age of 50.^8^ Laboratory Information Management Systems (LIMS) should be capable of supporting suitable substitutions while blocking the issue of inappropriate substitutions.

Age‐appropriate components should be used wherever available. In emergency situations, it may not be possible to meet all additional paediatric specifications. In these situations, the selection of substitutions should follow national guidance.^20^

Tracking blood components once they have been issued may be challenging, especially if sent to multiple areas. Consideration should be given to the use of paper logs or whiteboards to record the issue of blood components, shock packs or other deliveries.

Arrangements must be in place for the traceability of blood sent to other hospitals and the Ambulance Service.

Hospital Transfusion Laboratories should be able to provide details of blood and blood component usage following a Major Incident to NHSBT within 72 hours, to guide the management of patients and bloodstocks in relation to future events.

## 8 REGULATORY REQUIREMENTS

Transfusion support in the context of an incident should comply with legislation (BSQR, 2005) and best practice. Records should be retained for both regulatory and forensic purposes.

Due consideration must be given to securely maintaining the cold chain of any blood components stored and transported during an incident. Special care is required when Hospital Transfusion Laboratories move “stock” of blood components to treatment areas in a Major Incident.

Hospital Transfusion Laboratories should have protocols for the timely thawing and issue of plasma together with the option of post‐thaw storage of FFP at 4°C for up to 5 days.^21^

The use and disposal of any blood component must be documented in the clinical notes and in the Hospital Transfusion Laboratory records using the unique number of both the blood unit and the patient. These records must be kept for 30 years for compliance with the Blood Safety and Quality Regulations 2005.^22^

Hospital Transfusion Laboratories should have procedures for maintaining the systems for traceability of blood and blood components, used and wasted, in a Major Incident setting. Examples include the use of electronic systems or manual peel off tags attached to patient notes.

All adverse incidents related to either the provision of transfusion services and/or the use of blood components should be reported to the Hospital Transfusion Team. It is recognised that acute transfusion reactions may be difficult to diagnose during resuscitation of the critically ill.

## 9 RESILIENCE AND RECOVERY

### 9.1 *Staff support and welfare*

The welfare and well‐being of all staff during a Major Incident is highly important. During the event, the tasks required of staff may, by the very nature of the occurrence, be overwhelming. In addition, on the announcement of a Major Incident, there is often an immediate response from staff to assist. Therefore, there is a risk that staffing will be exhausted swiftly if a high percentage of members of staff attend immediately. Staff attendance should be managed.

Consequently, Hospital Transfusion Laboratories should have policies for the organisation of staff in a Major Incident with systems for provision of additional staff only if needed. Off duty staff should be advised to avoid coming into work until they are called in.

Trusts should consider having policies for providing food, rest facilities and accommodation for staff unable to travel home. Specific provision may be required for hospital transfusion staff unable to leave the laboratory area.

Major Incidents can be traumatic events, which cause stress, whatever their source or scale. Staff may need some psychosocial support in the time following the incident. In some circumstances, those affected may need additional support for a considerable period. Debriefing may help individuals and support the transfusion team.

At the command “Major Incident Stand Down” the transfusion team should hold a short “hot debrief” meeting drawing out issues that presented problems or where improvements can be made. Debriefs may also contribute to psychosocial support. Debrief should be repeated for each new shift and provided to individuals as required.

A representative from the transfusion department should attend their hospital hot debrief meeting which is normally initiated by the director leading the Gold control team. The time should normally be no later than a few hours after the incident.

Any documentation and experience from the incident should be used to capture lessons identified, and feedback to other stakeholders including NHSBT to support service improvement.

### 9.2 *Recovery phase from a major incident*

Response and recovery are not two discrete activities but may occur simultaneously. The recovery team should begin to plan recovery activities at the onset of the incident. As soon as the initial response phase is over, the focus should be on returning to “Business as Usual,” or normality, as soon as possible.

A Trust level recovery co‐ordinator should be appointed to co‐ordinate the response. The Transfusion Department is responsible for planning local recovery but will need to be aware of the wider Trust plan. The recovery strategy will normally cover some or all the key following objectives:

- Managing the return to normal service delivery
- Priority of elective services including the impact on targets
- Staffing levels in the immediate future
- Identify patients who require further surgical intervention or follow‐up arrangements following the incident
- Number of beds occupied by Major Incident casualties, including critical care beds and other specialist beds
- Support to staff welfare including appropriate counselling
- Re‐stocking of supplies and equipment including blood components
- Infrastructure and estate issues
- Auditing and reporting of the incident
- Communication, internally and externally, to core stakeholders
- Financial implications and financial recovery plan

Transfusion Laboratories should re‐assess their bloodstocks in the light of these future activities and adjust standing orders with NHSBT as required. Consideration should be given to the redistribution of short shelf‐life components within the Trust network or to other Trusts to prevent wastage due to time expiry.

Transfusion Laboratories should complete their traceability audits and endeavour to account for all blood components issued during the incident.

## 10 BUSINESS CONTINUITY

### 10.1 *Business continuity threats including cyber security*

Business Continuity is regarded as a separate process from Major Incident planning although it is recognised that there are potential overlaps. Business Continuity has its own strategy, alert and escalation process.

Local business continuity plans should be held in readiness in the Transfusion Laboratory as well as in the emergency planning and command control rooms. There are normally four levels of activation for a business continuity incident.

The main business continuity threats to Trusts which may impact the delivery of hospital‐based transfusion services include:

- Loss of staff due to illness such as pandemic flu or an infectious outbreak. Outbreaks may have a secondary impact with loss of staff due to childcare commitments or dependents' illness. Pandemic flu may have an impact on blood donation, resulting in blood shortage.
- Interruption to essential supplies other than blood. Examples may include blood products such as albumin and immunoglobulins. Departments should ensure that there is enough stock of critical consumables and be aware of the risks in the supply chain. Transfusion Laboratories may be required to work closely and proactively with pharmacy and other departments to ensure continuity of supply.
- Loss of building due to fire, bomb threat or contamination especially when dealing with potentially infective biological material. Systems should be in place to safely handle known infective samples and to decontaminate where required.
- Interruption to a utility, such as electricity, gas or water (drought). Specific consideration should be given to the maintenance of the cold chain. The cold chain may be compromised during hot weather if there is air‐conditioning failure.
- Interruption/loss of telephone or IT system. Modern clinical laboratory services, including transfusion services, are highly dependent on the Laboratory Information Management System for all aspects of blood banking.

Business Continuity plans should be in place to maintain essential services in the event of an IT failure. Hospital Transfusion Laboratories should maintain the capability to use manual techniques for testing and nonelectronic record keeping. Procedures should be in place to update IT records during the recovery phase.

It is specifically recommended that nonelectronic records of regularly transfused patients with clinically significant antibodies and special requirements are regularly maintained to enable timely transfusion in the event of a cyber‐attack or power failure.

Disaster planning and training should include cyber security, and the actions required to minimise impact. Trusts and pathology services should comply with cyber and data security good practice to reduce the risk of IT failure. Examples of current guidance and best practice are available via NHS Digital and the National Cyber Security Centre.

### 10.2 *Resilience and mutual aid*

Mutual aid is defined as an arrangement between Category 1 and Category 2 responders and other organisations not covered by the Civil Contingencies Act 2004.^1^ Mutual aid may occur within the same sector or across sectors and across boundaries, to provide and assist with additional resource during an emergency that may overwhelm the resources of a single organisation.

Where consideration is given to providing or requesting assistance from another NHS organisation, this should be agreed through local strategic command. The requirements, roles and responsibilities should be clearly set out in advance. Examples of mutual aid for transfusion might include movement of bloodstock and emergency use of fridges and freezers. NHSBT may be contacted through the Hospital Services department for mutual aid enquiries.

NHSBT aims to manage supplies of blood components and critical services to all customers during emergencies. Response to orders may be staged, and substitutions used where appropriate. Priority will be based on clinical need.

Emergency deliveries will be made in NHSBT liveried vehicles, usually directly to the transfusion laboratory. It may be necessary for a rendezvous point to be established when the security of a hospital is compromised. In such circumstances, an individual should be designated as the point of contact for the receipt of blood products.

Guidance for the arrangements for specialist chemical and biological antidote services is provided by NHS England.

## CONFLICT OF INTEREST

The authors declare no conflicts of interest.

## Supporting information


**Data S1**: Supporting Material

## APPENDIX 1

### MEMBERSHIP OF 2018 REVIEW GROUP

H.D. (Chair), Consultant in Transfusion Medicine, NHSBT; F.C., Consultant Haematologist, NHSBT & Imperial College Healthcare NHS Trust (Group Secretary); V Ameh, Consultant in Emergency Medicine, University of Manchester Medical School & The Royal Albert Edward Infirmary Wigan; N Batrick, Consultant in Emergency Medicine & Trauma Lead, Imperial College Healthcare NHS Trust; S Bassey Consultant Transfusion Scientist, Royal Cornwall Hospital; L Baxter, Specialist Practitioner of transfusion, Salford Royal Foundation Trust; P Bolton–Maggs, Transfusion Medicine SAC, Royal College of Pathologists; T Cowdrey, Business Continuity, NHSBT; S Glasgow, Clinical Research Fellow, The Centre for Trauma Sciences, Queen Mary University of London; A Jackson, Transfusion Practitioner United Lincolnshire Hospitals NHS Trust; S Robinson Consultant Haematologist, Guy's and St Thomas' NHS Foundation Trust; M Smith Consultant in Emergency Medicine, Salford Royal Foundation Trust; J Staves, Blood Transfusion Laboratory Manager, Oxford University Hospitals NHS Foundation Trust; Y Sorour, Chair of RTC Chairs, Barnsley Hospital NHS Foundation Trust; K Pendry, Consultant Haematologist, NHSBT & Central Manchester University Hospitals NHS Foundation Trust; J Uprichard, Consultant Haematologist, Chair of London Trauma & Haematology Group, St George's University Hospitals NHS Foundation Trust; A Weaver, Consultant in Emergency Medicine & Pre‐hospital Care, Clinical Director for Trauma, Bart's Health NHS Trust; C Wilkes, Regional Customer Service Manager NHSBT.

### MEMBERSHIP OF 2006 WRITING GROUP

A Cope (Chair), Department of Accident and Emergency, Peterborough Hospital; S Allard, Department of Haematology Bart's and the London NHS Trust & NHSBT; M Murphy, Department of Haematology, Oxford Radcliffe Hospitals NHS Trust and NHSBT; C, Kendrick, Department of Accident and Emergency, Peterborough Hospital; H Doughty, Department of Haematology, Birmingham NHS Foundation Trust and NHSBT; J Earley, Department of Haematology, Chelsea and Westminster Hospital; L Frith, NHSBT. Acknowledgments: Jane Pearson, Tissue Services, NHSBT.
