# Supplementary material for: Emergency preparedness, resilience and response guidance for UK hospital transfusion teams
Source: Transfus Med. 2020 Feb 4;30(3):177–85. doi: 10.1111/tme.12665 (PMC7317494; doi:10.1111/tme.12665)
Supplement: Supplementary file 1 — Data S1: Supporting Material [file TME-30-177-s001.docx]

SUPPORTING MATERIAL

Additional material may be found in the NBTC Emergency Planning Working Group section on the Joint United Kingdom (UK) Blood Transfusion and Tissue Transplantation Services Professional Advisory Committee website <https://www.transfusionguidelines.org/uk-transfusion-committees/national-blood-transfusion-committee/working-groups#Emergency>.
